# Correction: The forkhead transcription factor Foxj1 controls vertebrate olfactory cilia biogenesis and sensory neuron differentiation

**DOI:** 10.1371/journal.pbio.3003229

**Published:** 2025-06-10

**Authors:** Dheeraj Rayamajhi, Mert Ege, Kirill Ukhanov, Christa Ringers, Yiliu Zhang, Inyoung Jeong, Percival P. D’Gama, Summer Shijia Li, Mehmet Ilyas Cosacak, Caghan Kizil, Hae-Chul Park, Emre Yaksi, Jeffrey R. Martens, Steven L. Brody, Nathalie Jurisch-Yaksi, Sudipto Roy

The sixth author’s name is spelled incorrectly. The correct name is: Inyoung Jeong. The correct citation is: Rayamajhi D, Ege M, Ukhanov K, Ringers C, Zhang Y, Jeong I, et al. (2024) The forkhead transcription factor Foxj1 controls vertebrate olfactory cilia biogenesis and sensory neuron differentiation. PLoS Biol 22(1): e3002468. https://doi.org/10.1371/journal.pbio.3002468.
