# Supplementary figures and images for: The forkhead transcription factor Foxj1 controls vertebrate olfactory cilia biogenesis and sensory neuron differentiation
Source: PLoS Biol. 2024 Jan 25;22(1):e3002468. doi: 10.1371/journal.pbio.3002468 (PMC10810531; doi:10.1371/journal.pbio.3002468)

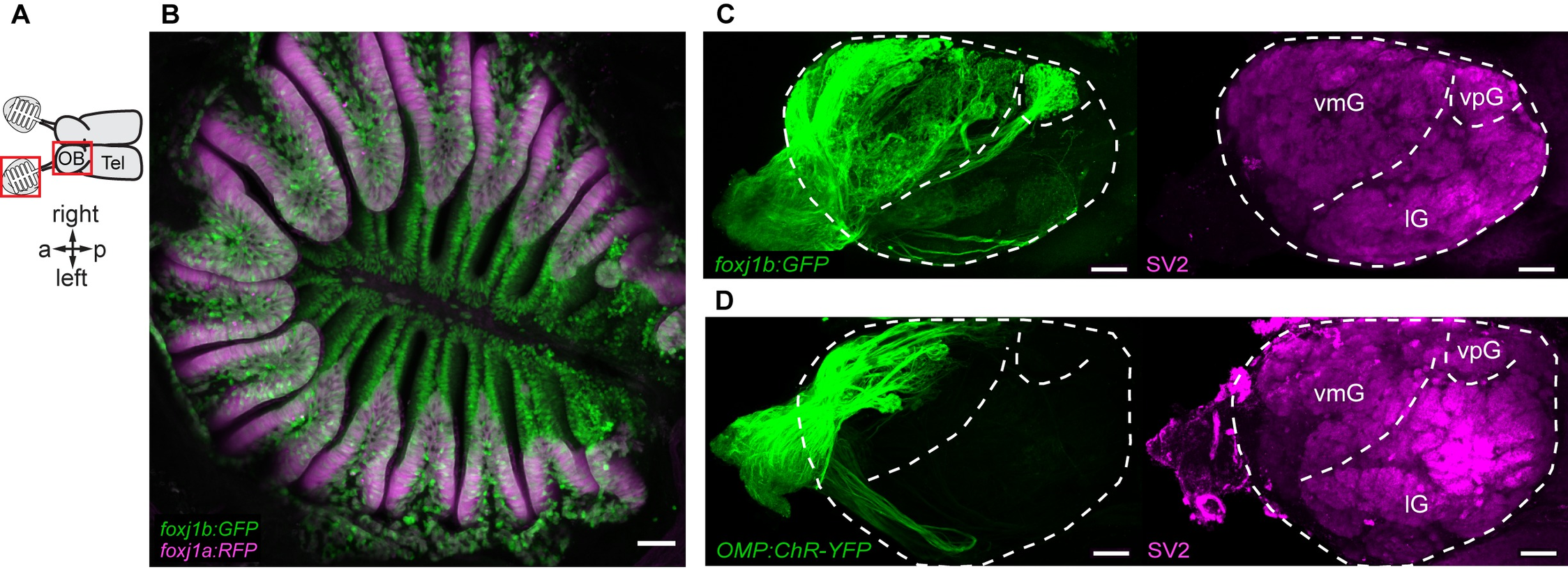

Supplement: S1 Fig — (A) Schematic showing the adult zebrafish OB connected to the OE. (B) Confocal image of an adult zebrafish OE showing expression of foxj1a (Gt(foxj1a:2A-TagRFP), magenta) and foxj1b (Gt(foxj1b:GFP), green). Note that foxj1a is mainly expressed at the tip of the lamellas where MCCs are located. Scale bar = 50 μm. (C, D) Projections of foxj1b- (C, Gt(foxj1b:GFP), green) and omp-positive OSNs (D, Tg(OMP:ChR-YFP) green) into the OB. Glomeruli are indicated by the presynaptic marker SV2. Note that foxj1b-expressing OSNs project to more glomeruli than omp-expressing OSNs. Scale-bars = 20 μm. a: anterior, p: posterior. Tel = Telencephalon. MCC, motile multiciliated cell; OB, olfactory bulb; OE, olfactory epithelium; OSN, olfactory sensory neuron. (TIF) [file pbio.3002468.s001.tif]

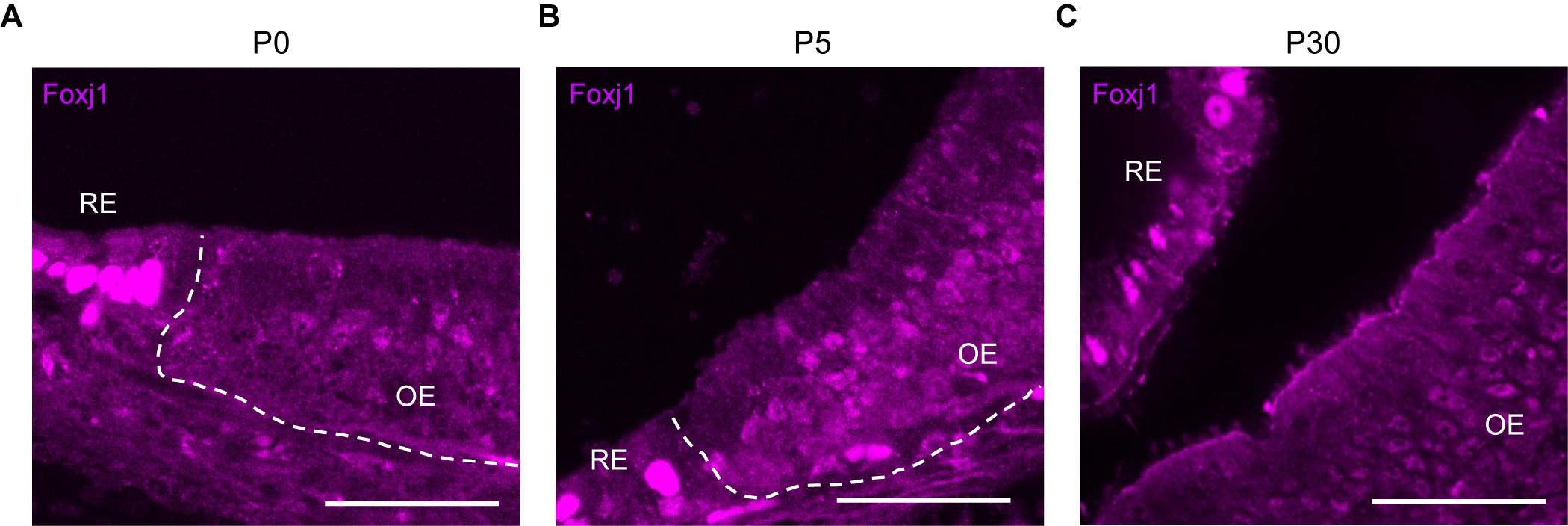

Supplement: S2 Fig — (A-C) Immunostaining of Foxj1 at different animal ages (newborn P0 (A), day 5 P5 (B), and adult P30 (C)) in OSNs of wild-type mice. Border between the OE and the RE is marked by a dashed line. Brightly labeled cells in the RE are respiratory MCCs. Scale bars = 50 μm. MCC, motile multiciliated cell; OE, olfactory epithelium; OSN, olfactory sensory neuron; RE, respiratory epithelium. (TIF) [file pbio.3002468.s002.tif]

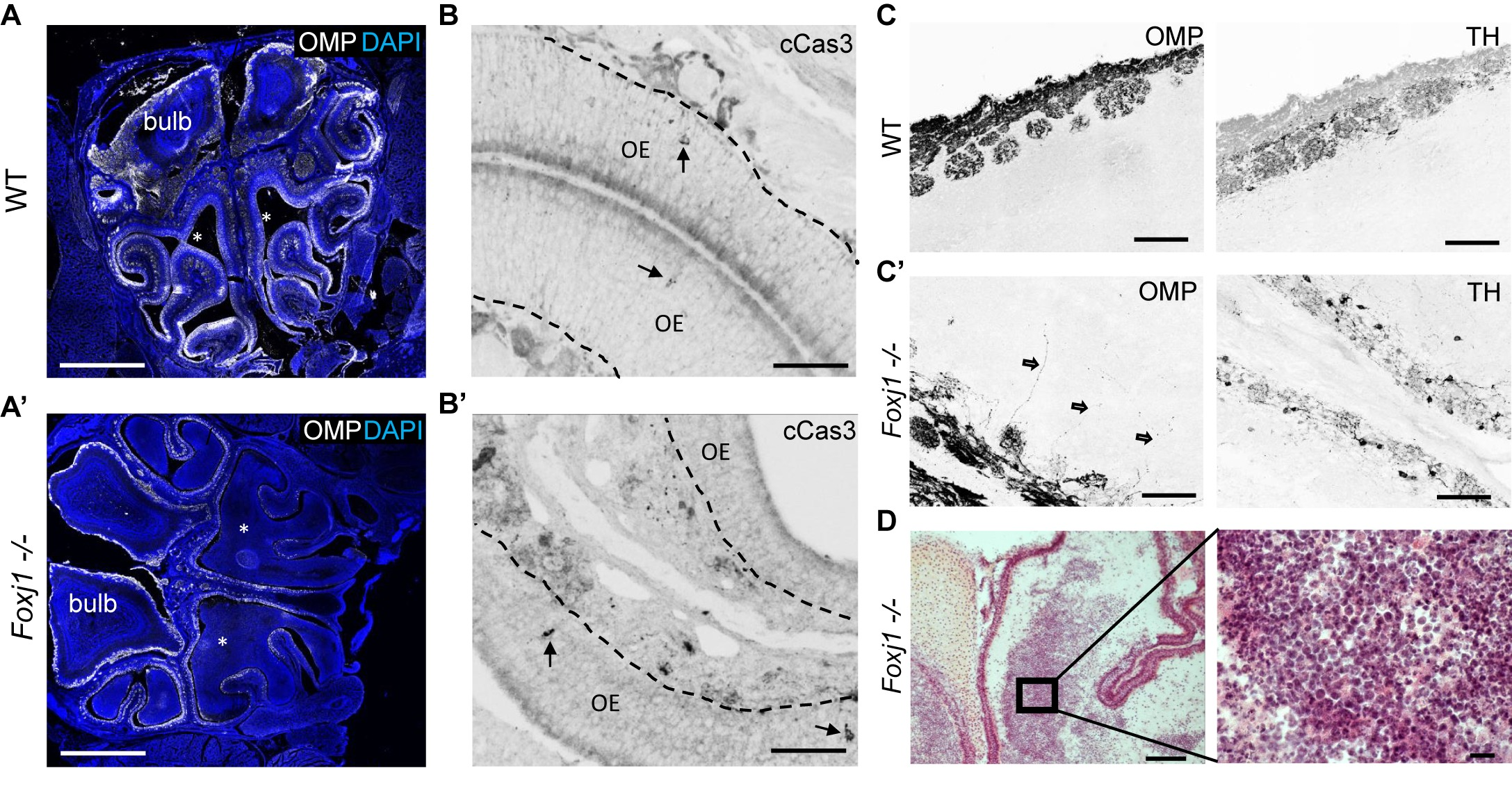

Supplement: S3 Fig — (A, A’) Zoomed out view of the nasal cavity of the WT and Foxj1−/− mouse. Nasal cavities are denoted by asterisks. (B, B’) Level of apoptosis in the OE was similarly low in the WT (B) and Foxj1−/− (B’) mouse (108 ± 24, n = 6, WT; 83 ± 7 cells per mms, n = 6, KO; 3 mice, p = 0.554) as determined by immunostaining for cleaved Caspase 3 (arrows). (C, C’) OMP immunostaining showed larger, strongly OMP-expressing glomeruli in OB of WT (C, top-left), but irregular, smaller glomeruli in Foxj1−/− mouse (C’, bottom-left). Wandering axons present in OB of Foxj1−/− mouse as axons did not fully converge within glomeruli overshooting in internal layers of the OB (C’, bottom-left, arrows). Similarly, TH expression denoting neural activity reduced in Foxj1−/− mouse (C’, bottom-right) showing weaker TH immunofluorescence (185.6 ± 19.7 a.u., n = 14, WT; 63.6 ± 7.9 a.u., n = 37, KO, 3 mice, p < 0.0001) when compared to WT mouse (C, top-right). (D) Nasal cavity of the Foxj1−/− mouse contained neutrophils as revealed by HE stain. Scale bar = 1,000 μm (A, A’), 100 μm (B, B’, D), and 10 μm (D, inset). HE, hematoxylin–eosin; KO, knockout; OB, olfactory bulb; OE, olfactory epithelium; OMP, olfactory marker protein; TH, tyrosine hydroxylase; WT, wild type. (TIF) [file pbio.3002468.s003.tif]

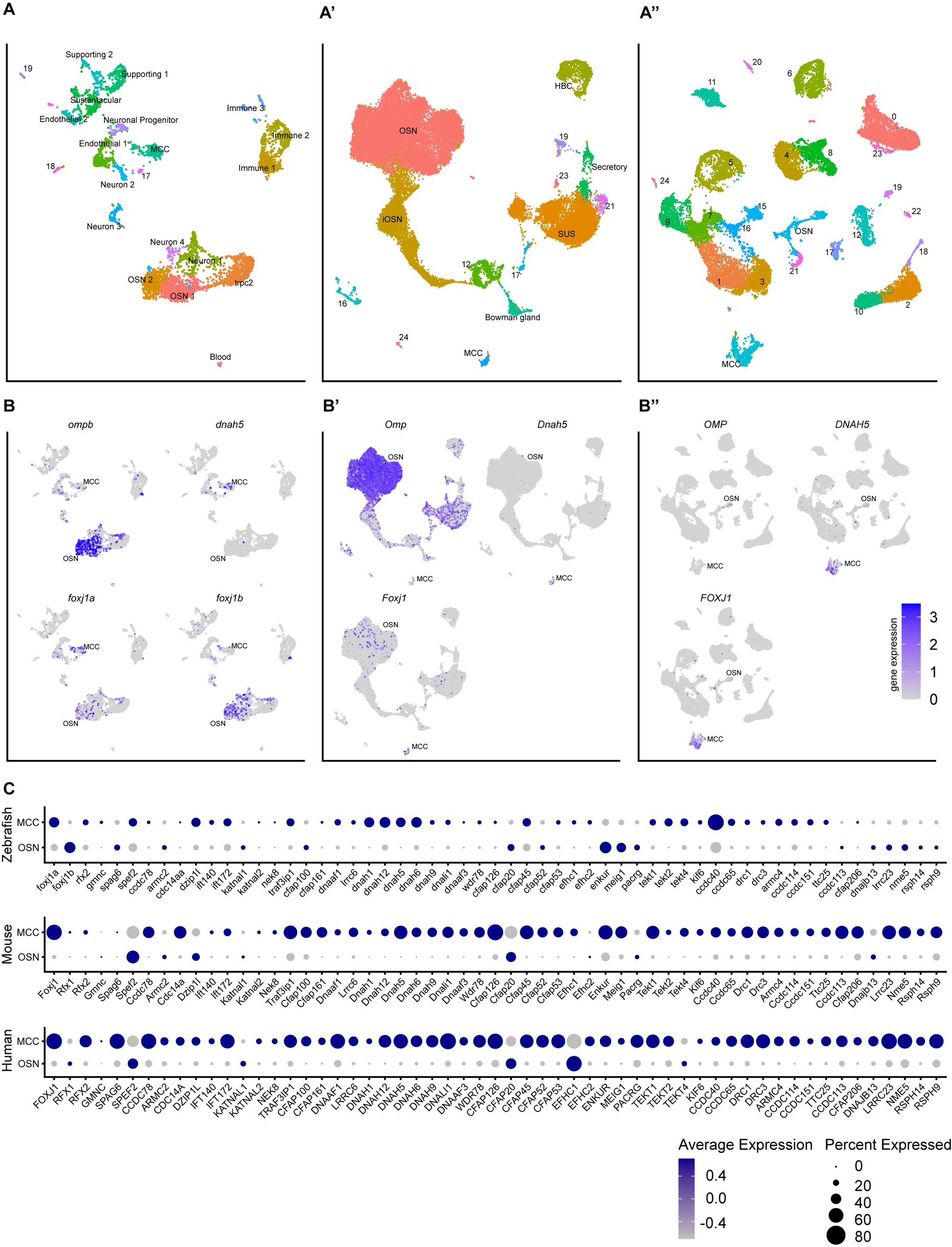

Supplement: S4 Fig — (A-A”) Dimensionality reduction plots of detected cell clusters on single-cell RNA sequencing data from zebrafish, mouse, and human olfactory epithelial tissue (calculated by umap). Cell clusters corresponding to MCCs and OSNs are shown. (B-B”) Marker genes omp, dnah5, and foxj1 expression across cell clusters. (C) Dot-plot showing expression levels of foxj1 target genes of interest in MCC and OSN cell clusters across species. MCC, motile multiciliated cell; OE, olfactory epithelium; OSN, olfactory sensory neuron. (TIF) [file pbio.3002468.s004.tif]
