# Supplementary material for: The forkhead transcription factor Foxj1 controls vertebrate olfactory cilia biogenesis and sensory neuron differentiation
Source: PLoS Biol. 2024 Jan 25;22(1):e3002468. doi: 10.1371/journal.pbio.3002468 (PMC10810531; doi:10.1371/journal.pbio.3002468)
Supplement: S2 Table — (DOCX) [file pbio.3002468.s007.docx]

| **REAGENT or RESOURCE** | **SOURCE** | **IDENTIFIER** |
| --- | --- | --- |
| **Antibodies** | | |
| SV2 antibody | Developmental Studies Hybridoma Bank | Cat# 2315387,  RRID:AB_2315387 |
| Anti-Acetylated Tubulin antibody, Mouse monoclonal | Sigma-Aldrich | Cat# T7451, RRID:AB_609894 |
| Rabbit anti-acetylated tubulin (Monoclonal) | Cell Signaling Technology | 5335S |
| Anti-Polyglutamylation Modification mAb (GT335) antibody | AdipoGen | Cat# AG-20B-0020, RRID:AB_2490210 |
| β-Catenin (D10A8) XP Rabbit mAb (Biotinylated) antibody | Cell Signaling Technology | Cat# 13727, RRID:AB_2798305 |
| Mouse HuC/D | Invitrogen | Cat# A-21271, RRID:AB_221448 |
| GFP Polyclonal antibody, Alexa Fluor 488 | Thermo Fisher Scientific | Cat# A-21311, RRID:[AB_221477](http://antibodyregistry.org/AB_221477) |
| Chicken anti-GFP, Polyclonal Antibody | Abcam | Ab13970 |
| Mouse anti-acetylated tubulin antibody (Monoclonal) | Sigma | T6793-100UL |
| Mouse anti-gamma tubulin antibody (Monoclonal) | Sigma | T6557-2ML |
| Goat anti-chicken IgG (H+L) Secondary Antibody, Alexa Fluor 488 | Invitrogen | REF# A11039 |
| Donkey anti-Mouse IgG (H+L) Secondary Antibody, Alexa Fluor 488 | Thermo Fisher Scientific | REF# A21202 |
| Donkey anti-Rabbit IgG (H+L) Secondary Antibody, Alexa Fluor 488 | Thermo Fisher Scientific | REF# A21206 |
| Goat anti-Rabbit IgG (H+L) Highly Cross-Adsorbed Secondary Antibody, Alexa Fluor 555 | Thermo Fisher Scientific | Cat# A-21429, RRID:AB_2535850 |
| Goat anti-Mouse IgG2b Cross-Adsorbed Secondary Antibody, Alexa Fluor 568 | Thermo Fisher Scientific | Cat# A-21144, RRID:AB_2535780 |
| Donkey anti-Mouse IgG (H+L) Secondary Antibody, Alexa Fluor 555 | Life Technologies | REF# A31570 |
| Goat anti-Mouse IgG2b Secondary Antibody, Alexa Fluor 633 | Thermo Fisher Scientific | Cat# A-21146, RRID:AB_2535782 |
| Anti-Acetylated Tubulin | Sigma-Aldrich | Cat# T6793 |
| Anti-Foxj1 mouse monoclonal | Thermo Fisher | Cat# 14-9965-82  RRID: AB_1548835 |
| Goat anti-OMP | Wako | Cat#  RRID |
| Chicken anti-Gap43 | EnCor Biotechnology | Cat# CPCA-GAP43, RRID:AB_2572284) |
| Rabbit anti-Ki67 | Novus | Cat# NB 500-170, RRID:AB_343263 |
| Rabbit anti-tyrosine hydroxylase | Millipore | Cat# AB152  RRID:AB_390204 |
| Rabbit anti-cleaved Caspase 3 | Cell Signaling | Cat# 9661L  RRID:AB_2341188 |
| Goat anti-Mouse IgG1-Alexa Fluor 546 | Thermo Fisher | Cat# A-21123  RRID: AB_2535765 |
| Donkey anti-Goat IgG (H+L) Cross-Adsorbed Secondary Antibody, Alexa Fluor 488 | Thermo Fisher | Cat # A-11055  AB_2534102 |
| Donkey anti-Mouse IgG (H+L) Highly Cross-Adsorbed Secondary Antibody, Alexa Fluor 647 | Thermos fisher | Cat # A-31571  RRID: AB_162542 |
| Donkey anti-Rabbit IgG (H+L) Highly Cross-Adsorbed Secondary Antibody, Alexa Fluor 594 | Thermos fisher | Cat# A-21207  RRID: AB_141637 |
| Hematoxylin and Eosin Stain Kit | Vector Laboratories | H-3502 |
|  |  |  |
| **Bacterial strains** | | |
| One Shot TOP10 Chemically Competent *E. coli* | Thermo Fisher | C404010 |
| **Chemicals, peptides, and recombinant proteins** | | |
| DAPI | Thermo Fisher Scientific | Cat# D1306; RRID:AB_2629482 |
| T7 RNA polymerase | Roche | 10881767001 |
| Ribonucleoside Triphosphate Set | Roche | 11277057001 |
| DNase I recombinant, RNase-free | Roche | 04716728001 |
| mMESSAGE mMACHINE T3 transcription kit | Thermo Fisher | AM1348 |
| mMESSAGE mMACHINE T7 transcription kit | Thermo Fisher | AM1344 |
| mMESSAGE mMACHINE SP6 transcription kit | Thermo Fisher | AM1340 |
| QIAGEN Plasmid Midi kit | QIAGEN | 12143 |
| PureYield Plasmid Miniprep System | Promega | A1223 |
| QIAprep Spin Miniprep kit (250) | QIAGEN | 27106 |
| XbaI | New England Biolabs | R0145S |
| BfuAI | New England Biolabs | R0701S |
| BspQI | New England Biolabs | R0712S |
| EcoRI | New England Biolabs | R3010L |
| NotI | New England Biolabs | R3189L |
| Trizol | ThermoFisher | Cat# 15596026 |
| Ambion Nuclease-free water | ThermoFisher | Cat# AM9932 |
| Formaldehyde solution (PFA) | Sigma | Cat# F8775/25ml |
| Triton X-100 | Merck | Cat# 1086031000 |
| Dimethyl sulfoxide (DMSO) | Sigma | Cat# D8418 |
| Bovine Serum Albumin (BSA) | PanReac AppliedChem | Cat# A1391 |
| Glycerol | VWR | Cat# 24387.292 |
| Phosphate Buffered Saline (PBS) | ThermoFisher | Cat# BR0014G |
| Trypsin | ThermoFisher | Cat# 27250018 |
| EDTA (0.5 M), pH 8.0, RNase-free | ThermoFisher | Cat# AM9260G |
| MS222 (Tricaine methanesulfonate) | Sigma-Aldrich | Cat# E10521 |
| LMP Agarose | Fischer Scientific | Cat# 16520100 |
| Fluorescein (free acid) | Sigma-Aldrich | Cat# F2456 |
| Alanine | Sigma-Aldrich | Cat# A7627 |
| Phenylalanine | Sigma-Aldrich | Cat# P5482 |
| Aspartic acid | Sigma-Aldrich | Cat# A5474 |
| Arginine | Sigma-Aldrich | Cat# A5006 |
| Methionine | Sigma-Aldrich | Cat# M9625 |
| Asparagine | Sigma-Aldrich | Cat# A0884 |
| Histidine | Sigma-Aldrich | Cat# H8125 |
| Taurodeoxycholic acid (TDCA) | Sigma-Aldrich | Cat# T0875 |
| Taurocholic acid (TCA) | Sigma-Aldrich | Cat# T4009 |
| Inosine monophosphate (IMP) | Sigma-Aldrich | Cat# I4625 |
| Adenosine monophosphate (AMP) | Sigma-Aldrich | Cat# A9396 |
| Zebrafeed <100µm | Sparos | N/A |
| x2 power up SYBR master mix | Thermo Fisher Scientific | Cat# A25742 |
| Microamp optical 96 well reaction plate (Applied biosystems) | Thermo Fisher Scientific | Cat# N8010560 |
| Acetone | VWR | Cat# 20066.296 |
| Proteinase K from tritirachium album | Sigma | Cat# P2308-25MG |
| Protector RNase Inhibitor | Roche | 03335399001 |
| 2x MyFi Mix | Meridian Bioscience | MFX-122202A |
| Sodium Hydroxide pellets | 1^st^ BASE | BIO-1530-1Kg |
| Tris-HCl | 1^st^ BASE | BIO-1500-1Kg |
| SSC Buffer 20x Concentrate | SIGMA | S6639-1L |
| **Critical commercial assays** | | |
| RNeasy mini kit | Qiagen | Cat# 74004 |
| RNase-free Dnase set | Qiagen | Cat# 79254 |
| **Deposited data** | | |
|  |  |  |
| **Experimental models: Zebrafish** | | |
| *Tg(elavl3:Gcamp6s)* | Ahrens lab, Janelia farm [84] | ZFIN: ZDB-ALT-141023-1 |
| *Gt(foxj1a:2A-TagRFP)^FRZCC 1100^* | Park lab, University of Korea | This study |
| *T2BGSZ10 Gt(foxj1b:GFP)* | Meng lab, Tsinghua University [33] | ZFIN: ZDB-ALT-110301-1 |
| *Tg(OMP:Gal4;UAS:NTR-mCherry)* | Yoshihara and Okamoto lab, Riken [34, 35] | ZFIN: ZDB-ALT-160921-4 and ZDB-ALT-110215-7 |
| *Tg(UAS:GCaMP6s)* | Kawakami lab, NIG [101] | ZFIN: ZDB-ALT-170615-4 |
| *Tg(OMP:ChR2-YFP)* | Yoshihara lab, Riken [25] |  |
| *foxj1a* mutant (foxj1a^nw3^) | Yaksi lab, NTNU [82] | ZFIN: ZDB-ALT-190620-14 |
| *foxj1a* mutant (foxj1a^sq5717^) | Roy lab, A*STAR [83] | ZFIN: ZDB-ALT-190529-1 |
| *foxj1b* mutant (foxj1b^sq5719^) | Roy lab, A*STAR [32] | ZFIN: ZDB-ALT-221221-12 |
| *Tg(trpc2b-GAL4)* | Raper lab, [36] | ZFIN: ZDB-ALT-140818-2 |
| *Tg(UAS-Ntr-mCherry)* | Halpern lab, [81] | ZFIN: ZDB-ALT-070316-1 |
| **Experimental models: Mouse** | | |
| *Foxj1* knockout | Steven Brody lab, Washington University | [102] |
| **Oligonucleotides** | | |
| *foxj1a* gRNA sequence  GGACCCCTGGCCGGAGGGCT | Macrogen | N/A |
| *foxj1a* gRNA sequence for gRNA synthesis  taatacgactcactataGGACCCCTGGCCGGAGGGCTgttttagagctagaaatagc | Macrogen | N/A |
| *foxj1a* genotyping forward primer (5’ junction forward)  CCTTAGCCCTACGCCTTCAAGATG | Macrogen | N/A |
| *foxj1a* genotyping reverse primer (3’ junction reverse)  AGGCTGTCGTCTAAATTGTCGGAG | Macrogen | N/A |
| RFP 5’ junction reverse primer CCTTAATCAGTTCCTCGCCCTTAGA | Macrogen [86, 87] | N/A |
| RFP 3’ junction forward primerCGACCTCCCTAGCAAACTGGGG | Macrogen [86, 87] | N/A |
| *foxj1a* 5’ homology arm forward sequence for cloning  gcggCCCTTGTCAGGACCATGCTCTCCATGAGTTCCATGGACCCCTGGCCGGAGGGC | Macrogen | N/A |
| *foxj1a* 5’ homology arm reverse sequence for cloning  atccGCCCTCCGGCCAGGGGTCCATGGAACTCATGGAGAGCATGGTCCTGACAAGGG | Macrogen | N/A |
| *foxj1a* 3’ homology arm forward sequence for cloning  aagAAACGGCCTGAGCCGCTGCAGTCACCACTTCCTCCTCCAAGCCCACCGAGC | Macrogen | N/A |
| *foxj1a* 3’ homology arm reverse sequence for cloning  cggGCTCGGTGGGCTTGGAGGAGGAAGTGGTGACTGCAGCGGCTCAGGCCGTTT | Macrogen | N/A |
| *foxj1a* CRISPR deletion check Forward primer  GAGCTAACCAAATCAATCGGGG | Integrated DNA Technologies (IDT) | N/A |
| *foxj1a* CRISPR deletion check Reverse primer  CGTAAGTTCGCGCAGGATTG | IDT | N/A |
| *foxj1a* CRISPR het/homo check Reverse primer  ATCCAGTAGAGCGTCCCAGT | IDT | N/A |
| *foxj1b* CRISPR deletion check Forward primer  CTCCATCCTCAGTGCCAACC | IDT | N/A |
| *foxj1b* CRISPR deletion check Reverse primer  TCTTCAGACCAGCAAAGACAGT | IDT | N/A |
| *foxj1b* CRISPR het/homo check Reverse primer  CGGCTCTGCGTATCTGTAGT | IDT | N/A |
| Forward and reverse primer sequences for *ompb* probe synthesis: Fwd – ATGATGCGTCTGCGCGTTCAG  Rev- CAGACTGACCAGAAGAGAGCCG | IDT | N/A |
| Forward and reverse primer sequences for *cnga4* probe synthesis: Fwd – ATGAACAAAAATCGCTGGACAAGGC  Rev - TATTGGCGTCTCATGGAGGCAAACT | IDT | N/A |
| **Recombinant DNA** | | |
| pT3TS-nls-zCas9-nls plasmid DNA | [89] | RRID: Addgene_46757 |
| pGTag-TagRFP-SV40 plasmid DNA | [86] | RRID: Addgene_117807 |
| **Software and algorithms** | | |
| ImageJ/Fiji | https://imagej.net/software/fiji/ | N/A |
| Seurat | https://satijalab.org/seurat/index.html  [92-95] | N/A |
| Matlab |  |  |
| GraphPad Prism 9.4.1 | https://www.graphpad.com/scientific-software/prism/ |  |
| Nikon NIS Elements 5.11 | https://www.microscope.healthcare.nikon.com/resources |  |
| RNA sequencing | [96-98] |  |
