# Supplementary material for: The forkhead transcription factor Foxj1 controls vertebrate olfactory cilia biogenesis and sensory neuron differentiation
Source: PLoS Biol. 2024 Jan 25;22(1):e3002468. doi: 10.1371/journal.pbio.3002468 (PMC10810531; doi:10.1371/journal.pbio.3002468)
Supplement: Attachment — Submitted filename: Response to Reviewers.docx [file pbio.3002468.s008.docx]

Reviewers' comments

Rev. 1:

The submitted manuscript explores a role for the transcription factor Foxj1 in olfactory sensory neuron cilia development in both zebrafish and mouse. The authors demonstrate a role for this protein in non-motile ciliated cells. They effectively demonstrate changes in OSN number in the mouse OE in Foxj1 knockouts, although claims that Foxj1 controls OSN-sepcific genes are not strongly backed up in the mouse. The reduction in both iOSNs and mOSNs, without increase in apoptotic cells, and decrease in Ki67 (proliferating) cells, raises questions of if Foxj1 is more functional in the early generation of OSNs. Single cell analysis of mOSNs from WT and KO mice would strengthen claims. Additionally the bulb phenotype in mouse would largely appear to be due the reduction in OSNs, which may show more wandering due to lower activity, though there is not sufficient data to fully support that. Functional studies in zebrafish strengthen the manuscript however, and provide compelling data the mOSN function in that species is altered specifically. On the whole, the manuscript is largely well written, and the results are interesting. This manuscript would add to our understanding of cilia development in OSNs which are critical for a functional olfactory system.

Our response: It is definitely an interesting possibility that Foxj1 is more active in earlier developmental stages. A scRNAseq would be very instrumental in answering this question, but goes beyond the scope of the present study. In any case, we have now acknowledged the reviewer’s comment in the text (discussion).

Some issues needing clarification

1) Figure 1: What ages are representative images from in panels L, M and N?

Our response: Mouse age was P30 (panels L and N), and P21 (panel M). This has now been indicated in the legends.

2) Figure 2: Not clear in the panels/legend what age the mouse sections are from. Schematics are where slices come from would be helpful. For example, images in F are clearly from different regions of the OE. In H, representative glomeruli images come from a more dorsal or lateral (WT) side and medial (KO) edges. Glomeruli have different sizes across regions of the bulb, showing images from the same region would allow better comparisons.

Our response: Mice were used at 21 day of age. This information has been added to the legend. Now, representative images used in panel F and H represent similar areas of the mouse OE.

3) In the figure legends, it is not always clear how many animals were used for different immunolabeling experiments. The n appears to related to slides/sections, glomeruli counted and animals depending on which legend, however number of animals is not consistently reported.

Our response: The number of animals in each experiment has been updated.

4) This statement should be reworded "In case of mice, loss of Foxj1 in P21 pups resulted in a severe disfigurement of the olfactory system at the gross anatomical level, manifest in largely undeveloped turbinates and the entire nasal cavity was filled with DAPI-positive cellular masses composed of neutrophils (S3A, SA' and SD Figs)." As written is suggests that Foxj1 was selectively targeted in P21 mice, and not analysis of Foxj1 loss on OE anatomy using P21 aged mice.

Our response: The statement has been reworded to reflect the animal age and avoid ambiguity.

5) The statement that "aberrant innervation also resulted in smaller size of glomeruli" is not supported by data. Smaller size is most likely linked to significant reduction in both immature and mature OSNs.

Our response: We agree with the critique, and the statement was removed from the text. The reviewer comment has been added to the text.

6) A secondary marker for glomeruli (GAP43, or M/T dendrite marker) should be used to confirm that regions lacking OMP are actually glomeruli.

Our response: ‘’Empty’’ glomeruli was a curious finding, not really adding to the main story. We have now removed this observation from the text.

Rev. 2:

In their manuscript entitled "Vertebrate olfactory cilia and sensory neuron differentiation is programmed by the forkhead domain transcription factor Foxj1", Dheeraj Rayamajhi and coworkers show that the forkhead domain-containing transcription factor (FoxJ1), known to be involved in the activation of ciliome genes in motile cilia cell-types, is also involved in the biogenesis of immotile olfactory cilia and the development of the olfactory receptor neurons (ORNs).

To obtain their results, the authors employed whole-mount in-situ hybridizations, hybridization chain reactions, RNA sequencing techniques, In-vivo confocal imaging, immunostainings, etc.

The study is very well conducted, the logic of the experiments is fine, the methodology is sound, the statistical analysis is well done, and the authors provide convincing evidence for all their claims.

The results are extremely interesting for individuals working in the field of olfaction but also for people working in the field of ciliary physiology and for biologists in general. The obtained results are, without a doubt, a significant advance in the relevant fields. It was a pleasure to read the manuscript.

However, I feel that before publication, a few points (see below) must be clarified and better explained. I think it is necessary to discuss some of the obtained results better (see my comments below).

Specific critique:

In the Introduction, you should better explain the differences between the olfactory systems of zebrafish, mice (and humans). You do not mention that several different ORN-types, ciliated and non-ciliated, exist in zebrafish. Adding this information will help the readers to understand the study better.

Our response: We have discussed the different kinds of zebrafish OSNs in the first results section as we feel this is the appropriate place to discuss this issue.

In Figure 1 E and Figure H-J: Not all foxj1b-expressing cilia/cells are Gαolf-positive. Could you speculate about the identity of the additional foxj1b expressing (ciliated) ORNs? Also, is it known which morphologic ORN-type innervates the ventral medial glomeruli? I did not find this information in the manuscript.

Our response: We have observed a subpopulation of *foxj1b* expressing cells that are not Gαolf-positive. These cells were shown to project to distinct glomeruli, such as the mdG as shown at the larval stage (Figure 1 H-I) and the vmG and vpG at the adult stage (Figure S1 C). We have investigated the identity of this group of cells by screening the literature on zebrafish olfactory bulb glomerular identities. These particular glomeruli have been shown to be negative for the markers for ciliated ONSs (Calretinin & Gαolf) and for the marker for Crypt cells (S100), both in the larval (Braubach et al, 2013, DOI:10.1523/JNEUROSCI.5185-12.2013) and adult (Braubach et al, 2012, DOI:10.1002/cne.23075) stages. There was also no overlap with the kappe neurons, which are shown to project to a single target glomerulus among the mdG2 (Ahuja et al, 2014, DOI:10.1038/srep04037). We expect these cells to have a primary cilium, but whether they house specialized cilia similar to that of the ciliated OSNs remains to be answered.

Figure 1 M: There are still some puncta in this figure. What could this puncta possibly be?

Our response: Foxj1 is expressed in multiciliated cells, which are either sensory neurons located in the OE or cells carrying motile multicilia in the RE. The residual puncta detected in the knock-out tissue may represent non-specific staining of blood vessels or mesenchymal tissue located in the lamina propria under the basement membrane. We have now clarified this in the legend.

Figure 2A: I do not see a big difference between the stainings in foxj1b mutant and foxj1a/b double mutant larvae. In the text that describes these results, you claim that there is a difference. Did you do any statistics? Could you please explain what you mean here?

Our response: We thank the reviewer for bringing up this point. We agree that the difference between the *foxj1b* mutant and the *foxj1a/b* double mutant is not as easy to see. Unfortunately, we were unable to do statistical analyses on the images due to limitations in acquisition. We have removed the sentences that implied this difference between *foxj1b-/-* and *foxj1a-/-;foxj1b-/-* embryos. Nevertheless, we also provide here additional confocal images of the two datasets, where the cilia are labelled with the anti-acetylated tubulin marker. Anti-glutamylated tubulin marker, labelling the bases of MCC cilia, is also included to better differentiate the cilia of ORNs in the *foxj1b* mutant images. As you can appreciate, it is possible to identify intact cilia on the ORNs in the *foxj1b* mutant animals (indicated by white arrows). This was observed in the majority (4 out of 6) of *foxj1b* mutant animals in the dataset (n = 6). In contrast, all cilia were lost in all double mutants (n = 6). Unfortunately, the anti-acetylated tubulin antibody labels not only the cilia but also the dendrite of the ORN, making it difficult to distinguish between the two easily. (Scale bars: 10μm)

In Figure 2B, you show only a small part of the OB. Why do you not show the whole OB? How is the innervation (Gαolf-positive ORNs) in the rest of the OB?

Our response: We observed no innervation of Gαolf-positive ORNs to the rest of the OB at this developmental stage. Uncropped images showing the entire OB for the control and double mutant fish are provided below.

Figures 2E, F, G: How can you tell that in mutant mice, the apical ciliary layer of the OE was disrupted entirely? I cannot see/identify cilia in the wild-type mice in the figures. Also, how do you explain that some ORNs are still present in the OE of mutant mice? How can these ORNs be formed? How do these ORNs differ from those that are not formed? Better discuss.

Our response: We agree with the reviewer. Using chemical fixation, it is really hard to preserve the intact ciliary layer. If live animals were available, *en face* imaging would directly answer the question. However, while we cannot resolve individual cilia, the presence or absence of a prominent band of acetylated tubulin staining at the apical surface is a strong indication of ORN cilia. Our previous work demonstrated that intact cilia are not necessary for OSN survival (McIntyre et al., 2015; Williams et al., 2016) so it is possible that these neurons form without cilia or with dramatically shorter or deformed cilia.

Figure 2H: Some glomeruli completely lacked innervation by OMP-positive ORN-axons. You state that this suggests aberrant targeting by the ORNs. Why aberrant targeting? Aren't these glomeruli "empty" because the ORNs that innervate them are missing in the mutants? Did you do any quantification of the glomeruli data?

Our response: As answered to the reviewer #1, identifying these cavitations in the tissue as glomeruli is perhaps the wrong nomenclature. We have removed this language and clarified the issue in the text.

Figure 3A: You should state in the results that describe these data that these data come from wild-type animals.

Our response: We thank the reviewer for this comment and have updated the text accordingly.

Odorant-stimulation data: You show that in foxj1b mutant zebrafish, but not in foxj1a mutants, the formation of olfactory cilia is strongly reduced (Figure 2A). In Figure 2A, I do not see an apparent difference in the the ciliary signal between foxj1b and foxj1a/foxj1b mutants (see also my comment above). How does this fit together with your odorant stimulation data? How can the responses to bile acids in foxj1b mutants not be impaired but be impaired in double mutant zebrafish larvae? Please better explain and discuss this observation.

Our response: We acknowledge this difference of responses between the *foxj1b* mutant and double mutant fish brought up by the reviewer. The two paralogous genes, *foxj1a* and *foxj1b* are not completely exclusive to MCCs and OSNs, respectively. As shown through immunohistochemistry in Figure 1C and the single-cell RNA sequencing analysis in Figure 3A, there is a population of OSNs that are also *foxj1a* positive. We speculate the presence of the wild-type *foxj1a* gene to have a compensatory effect on ciliated OSN activity, making the olfactory phenotype not as stark in the *foxj1b* mutants compared to the double mutants. We also show that the double mutants have a significant reduction in the size of the nose (Figure 2D), potentially due to external factors such as impacted flow patterns during the development of the olfactory epithelium, which we suspect might be further exacerbating the olfactory phenotype in the double mutants.
